# Hypoxia-Induced Changes in the Bioactivity of Cytotrophoblast-Derived Exosomes

**DOI:** 10.1371/journal.pone.0079636

**Published:** 2013-11-11

**Authors:** Carlos Salomon, Miharu Kobayashi, Keith Ashman, Luis Sobrevia, Murray D. Mitchell, Gregory E. Rice

**Affiliations:** 1 Centre for Clinical Diagnostics, University of Queensland Centre for Clinical Research, Brisbane, Australia; 2 Cellular and Molecular Physiology Laboratory (CMPL), Division of Obstetrics and Gynaecology, School of Medicine, Faculty of Medicine, Pontificia Universidad Católica de Chile, Santiago, Chile; VU University Medical Center, Netherlands

## Abstract

Migration of extravillous trophoblasts (EVT) into decidua and myometrium is a critical process in the conversion of maternal spiral arterioles and establishing placenta perfusion. EVT migration is affected by cell-to-cell communication and oxygen tension. While the release of exosomes from placental cells has been identified as a significant pathway in materno-fetal communication, the role of placental-derived exosomes in placentation has yet to be established. The aim of this study was to establish the effect of oxygen tension on the release and bioactivity of cytotrophoblast (CT)-derived exosomes on EVT invasion and proliferation. CT were isolated from first trimester fetal tissue (n = 12) using a trypsin-deoxyribonuclease-dispase/Percoll method. CT were cultured under 8%, 3% or 1% O2 for 48 h. Exosomes from CT-conditioned media were isolated by differential and buoyant density centrifugation. The effect of oxygen tension on exosome release (µg exosomal protein/10^6^cells/48 h) and bioactivity were established. HTR-8/SVneo (EVT) were used as target cells to establish the effect (bioactivity) of exosomes on invasion and proliferation as assessed by real-time, live-cell imaging (Incucyte™). The release and bioactivity of CT-derived exosomes were inversely correlated with oxygen tension (p<0.001). Under low oxygen tensions (*i.e.* 1% O2), CT-derived exosomes promoted EVT invasion and proliferation. Proteomic analysis of exosomes identified oxygen-dependent changes in protein content. We propose that in response to changes in oxygen tension, CTs modify the bioactivity of exosomes, thereby, regulating EVT phenotype. Exosomal induction of EVT migration may represent a normal process of placentation and/or an adaptive response to placental hypoxia.

## Introduction

Placentation is an oxygen sensitive process. The events that occur from the time of implantation to maternal perfusion of the placenta are influenced and directed by site-specific oxygen tensions [1]. At the time of embryo implantation, the intrauterine oxygen tension is ∼3% [2] while the decidua and myometrium oxygen tension is ∼8–12% [3]. This standing oxygen gradient is thought to promote and direct the invasion of extravillous trophoblast cells (EVT) into the decidua and myometrium where they engage and remodel maternal spiral arterioles [4], [5]. Intraluminal EVT occludes spiral arterioles to maintain a low oxygen tension environment that is requisite for normal early placental and fetal development. Towards the end of the first trimester, low resistance, high capacity flow is restored and the placental intravillous space is perfused with the maternal blood thus establishing effective materno-fetal exchange.

Factors that compromize trophobast cell (both cytotrophoblast (CT) and EVT) function during this critical period of development may dramatically effect subsequent fetal growth, outcome of pregnancy and the life-long disease risk profile of the newborn [6], [7]. EVT migration is affected by oxygen tension [8], [9]. Perturbation of intrauterine oxygen tensions, therefore, may compromise normal placental development. The molecular mechanisms by which oxygen tension regulates EVT function and cell-to-cell communication with maternal tissues remain to be fully elucidated.

Recently, the role of exosomes in cell-to-cell communication has been established [10]–[13]. Exosomes are nanoparticles (40–100 nm) membrane vesicles that are released following the exocytotic fusion of multi-vesicular bodies with the cell membrane[14]. They have been identified in plasma under both normal and pathological conditions [15] and their concentration increases with disease severity and/or progression [16], and in response to oxidative stress [17].

Recently, we demonstrated that exosomes are released from first trimester placental mesenchymal stem cells (pMSC) and increases endothelial cell migration and vascular tube formation [10]. In addition, the release of exosomes from pMSC was increased under low oxygen tension. These data are consistent with the hypothesis that in response to changes in the environmental milieu (such as oxygen tension) placental cells release exosomes that modify the phenotype of recipient cells. The role of cytotrophoblast cell-derived exosome in cell-to-cell communication and, in particular, their effect on EVT has yet to be established. Similarly, the effect of oxygen tension on the release and bioactivity of cytotrophoblast exosomes is not known.

The aim of this study was to test the hypotheses that: (*i*) exosomes released by cytotrophoblast cells (CT) increase EVT proliferation and invasion; and (*ii*) the bioactivity and protein content of CT-derived exosomes is altered by oxygen tension. An in vitro treatment/control experimental design was used to test these hypotheses. Exosomes were isolated from primary first trimester placental villous CT. A first trimester EVT cell line (HTR-8/SVneo) was used to assess the effect of CT-derived exosomes on cell proliferation, invasion and bioactivity. The data obtained in this study are consistent with the hypothesis that exosomes from first trimester CT promote HTR-8/SVneo invasion and proliferation, and exosomal protein content is oxygen tension dependent.

## Material and Methods

### First trimester sample collection

Tissue collection was approved by the Human Research Ethics Committees of the Royal Brisbane and Women's Hospital, and the University of Queensland (HREC/09/QRBW/14). Written informed consent was obtained from women for the use of placental tissue for research purposes after clinically indicated termination of pregnancy in compliance with national research guidelines. All experimental procedures were conducted within an ISO17025 accredited (National Association of Testing Authorities, Australia) research facility. All data were recorded within a 21 CRF part 11 compliant electronic laboratory notebook (Irisnote, Redwood City, CA, USA).

### Isolation of cytotrophoblast cells

First-trimester cytotrophoblast cells (CTs) were isolated from placentae (8–12 weeks) derived by the legal termination of pregnancy (n = 6 biological samples and 2 independent duplicate cultures per placenta) as previously described [18]. Briefly, 10–20 g of chorionic villi were washed in phosphate buffer saline (PBS) and were subjected to three sequential treatments with digestion buffer (0.25% trypsin (Gibco® Trypsin, Life Technologies™, Carlsbad, CA) and 0.1 mg of DNase I (Sigma-Aldrich™, Saint Louis, USA) per ml in HBSS (1x) + HEPES (25 mM). After each 15 min step, the supernatant fluid was layered over fetal calf serum (25 ml supernatant over 5 ml FCS). The supernatant fluid was centrifuged at 400× g for 10 min and the cell pellet was suspended in Dulbecco's modified Eagle's medium (DMEM) (Life Technologies™, Carlsbad, CA). A total of 6 ml of DMEM containing cytotrophoblast cells were layered onto a pre-formed colloidal silica gradient (Percoll, 10–70% (GE Healthcare Life Sciences, Piscataway NY, USA) diluted in HBSS 1X (Life Technologies™, Carlsbad, CA) and centrifuged at 500× g for 30 min. Cytotrophoblast cells displayed a buoyant density of 1.048 to 1.062 g/ml. Cells were collected and washed in 30 ml of DMEM plus 5 ml of FCS. CTs were further isolated by negative selection for monoclonal anti-HLA-A, B, C (clone W6/32, Accurate Chemical and Scientific Corp, Westbury, NY, USA) and negative selection for monoclonal anti-CD45 (Sigma-Aldrich) using magnetic beads (Dynabeads M450, Life Technologies) and characterized by specific markers: anti-cytokeratin-7 (clone 7H8C4, Sigma-Aldrich) and anti-vimentin (clone V9, Sigma-Aldrich).

### Isolation and characterization of cytotrophoblast-derived exosomes

#### Isolation

Exosomes were isolated from CT-conditioned incubation medium as previously described [10], [19]. In brief, CT-conditioned media was centrifuged at 300× g for 15 min, 2,000× g for 30 min, and 12,000× g for 45 min to remove whole cells, and debris. The resultant supernatant fluid was passed through a 0.22 µm sterile filter (Steritop™, Millipore, Billerica, MA, USA) and then centrifuged at 100,000× g for 75 min (Thermo Fisher Scientific Ins., Asheville, NC, USA, Sorvall, SureSpinTM 630/36, Tube angle: 900). The pellet was resuspended in PBS, washed and re-centrifuged (100,000× g for 75 minutes). The pellet was resuspended in PBS, layered on a continuous sucrose gradient (0.25–2 mM sucrose) (Sigma-Aldrich) prepared using a Hoefer SG30 gradient maker (GE Healthcare, NSW, Australia) and centrifuged at 110,000 g for 20 h. Fractions (10 in total) were collected with an 18-G needle and the density of each fraction was determined using the refraction index with OPTi digital refractometer (Bellingham+Stanley Inc., Lawrenceville, GA, USA). Fraction were diluted in PBS, and then centrifuged at 110,000× g for 70 min. Recovered exosomes pellet was resuspended in 50 µl PBS and their protein concentration determined by the dye-binding assay [19].

#### Exosome protein quantification

Exosome-containing fractions (1.146 to 1.199 g/ml) from sucrose gradients were combined in a single tube and centrifuged at 110,000× g for 70 min. Exosome pellets were resuspended in 50 µl PBS and their proteins determined by the DC™ Protein Assay (BIO-RAD, Hercules, CA, USA). Briefly, exosome samples (5 µl) were prepared by adding RIPA buffer (50 mM Tris, 1% Triton ×100, 0.1% SDS, 0.5% DOC, 1 mM EDTA, 150 mM NaCl, protease inhibitor) directly to exosomes suspended in PBS and sonicated at 37°C for 15 s three times to lyse exosome membrane and solubilize the proteins. Bovine serum albumin (BSA) diluted in RIPA buffer and PBS mixture (1∶1) was prepared as protein standards (0, 200, 400, 600, 800, 1,000, 1,500 µg/mL). Standards and samples (exosomes) were transferred to 96-well plates. Alkaline copper tartrate solution (BIO-RAD, USA) and dilute Folin Reagent (BIO-RAD, USA) were added to the samples and incubated for 15 min. The absorbance was read at 750 nm with the Paradigm Detection Platform (Beckman Coulter, USA).

#### Western Blot

Protein from each sucrose gradient fraction (10 in total) obtained after exosome isolation were separated by polyacrylamide gel electrophoresis, transferred to Immobilon-®FL polyvinylidene difluoride membranes (Millipore, Billerica, MA, USA) and probed with primary mouse monoclonal anti-CD63 (1∶2000; ab8219, Abcam, Sapphire Bioscience Pty Ltd, NSW, Australia), anti-CD81 (1∶1500, MAB6435, Abnova, Tapei City, Taiwan), anti-CD9 (1∶1,500; ab2215, Abcam, Sapphire Bioscience) and anti- Placental Alkaline Phosphatase (PLAP; 1∶1000; ab96588, Abcam, Sapphire Bioscience), as previously described [19], [20] exosome specific markers. Membranes were washed in Tris buffer saline and incubated (1 h) in TBST/0.2% BSA containing horseradish peroxidase– conjugated goat anti-mouse antibody. Proteins were detected by enhanced chemiluminescence with using a SRX-101A Tabletop Processor (Konica Minolta, Ramsey, NJ, USA). Bands densitometry ratio was determined using the GS-800 Calibrated Densitometer (Bio-Rad Laboratories, Hercules, CA, USA).

#### Transmission electron microscopy

Exosomes isolated by differential and continuous sucrose gradient centrifugation were analyzed by transmission electron microscopy. Exosome pellets (as described above) were fixed in 3% (w/v) glutaraldehyde and 2% paraformaldehyde in cacodylate buffer, pH 7.3. 5 µl of sample was then applied to a continuous carbon grid and negatively stained with 2% uranyl acetate. The samples were examined in an FEI Tecnai 12 transmission electron microscope (FEI™, Hillsboro, Oregon, USA).

### Effect of oxygen tension on exosome release

The effects of oxygen tension on the release of exosomes from CTs were assessed by incubating cells for 48 h in exosome-free culture medium (culture media was depleted of the contaminating exosomes using the same protocol for exosome isolation described previously and exosome-free culture media medium was confirmed by electron microscope) under an atmosphere of 5% CO2-balanced N2 to obtain 1%, 3% or 8% O2 (pO2 ∼6.75, ∼20.25 or ∼54 mmHg, respectively, n = 6 biological replicates in duplicate) in an automated PROOX 110-scaled hypoxia chamber (BioSpherics™, Lacona, NY, USA). The three-compartment hypoxia chamber allowed the simultaneous analysis of CT-cells from individual placentae thus reducing inter-assay variation. Cell viability was determined by Trypan Blue exclusion and Countess® Automated cell counter (Life Technologies™). In all experiments, viability remained at >95% and was not significantly different between groups (p = 0.85). Incubation media pO2 and pH were independently confirmed using a blood gas analyzer (Radiometer®, Brønshøj, Denmark) and NeoFox oxygen probe (Ocean Optics ™, Dunedin, FL, USA).

### Effect of exosomes on extra-villous trophoblast cell invasion and proliferation

A first trimester cell line (HTR-8/SVneo) was used to establish the effects of CT-derived exosomes on cell invasion. HTR-8/SVneo cells were kindly donated by Dr Charles H. Graham (Queen's University, Ontario, Canada) [21], [22]. The cells were cultured in RPMI-1640 (HyClone, South Logan, USA) supplemented with 10% FCS, 100 U/ml penicillin, and 100 µg/ml streptomycin (HyClone), at 37°C and 5% CO2. Before experiments, HTR-8/SVneo cells were cultured in RPMI-1640 supplemented with 0.2% FBS in 96-well culture plate (Corning Life Science, Tewksbury, MA, USA) according to the manufacturer's instructions for 18–24 h. During experiments, cells were visualized using a real-time cell imaging system (IncuCyte™ live-cell ESSEN BioScience Inc, Ann Arbor, Michigan, USA) and were imaged every 1–2 h to monitor treatment-induced effects on cell invasion. Time course and dose response effects were established using exosomes released from CTs incubated under 1% O2 for up to 48 h.

### Time Course

To determine the effects of CT-derived exosomes on cell invasion, HTR-8/SVneo cells were incubated in the presence (treatment: 100 µg exosomal protein/ml) or absence (control) of exosomes for up to 24 h (n = 12). Cell invasion was monitored by scratch assays [10]. A scratch was made on confluent monolayers using a 96-pin WoundMaker™ (BioScience Inc, Ann Arbor, Michigan, USA) and layered with 3 mg/ml collagen type I (Life Technologies™, Carlsbad, CA) and incubated at 37°C for 30 min to create a 3D matrix-gel. Wound images were automatically acquired and registered by the IncuCyteTM software system. CellPlayer™ 96-Well Invasion Assay software was use to fully automate data collection. Data were processed and analyzed using IncuCyte™ 96-Well Cell Invasion Software Application Module. Data are presented as the Relative Wound Density (Eizen, v1.0 algorithm). The rate of wound closure was compared using the half-maximal stimulatory time (ST50) and area under the time course curve (AUC)[10].

### Dose Response

To assess the effect of exosome concentration on extra-villous trophoblast invasion and proliferation, HTR-8/SVneo cells were cultured in the presence of increasing exosomes concentration (5, 10, 20, 50 and 100 µg exosomal protein/ml) for 24 h. Data are presented as half-maximal effective concentration (EC50).

### Effects of oxygen tension on CT-derived exosome bioactivity

To determine the effect of oxygen tension on exosome bioactivity, exosomes were obtained from CTs incubated under 1%, 3% and 8% O2 (as detailed above) for 24 h. The effects of exosomes on HTR-8/SVneo cell invasion and proliferation were assessed as detailed above.

### Proliferation

Cell proliferation was measured by the change in confluence in the presence of exosomes (100 µg exosomal protein/ml) isolated from 1%, 3% and 8% O2 using a standard confluence algorithm (IncuCyte™) as we have previously published[10], [23].

### Proteomic analysis of cytotrophoblast derived-exosomes by mass spectrometry (MS)

Isolated exosomes were solubilized in 8 M urea in 50 mM ammonium bicarbonate, pH 8.5, and reduced with DTT for 1 h. Proteins were then alkylated in 10 mM iodoacetic acid (IAA) for 1 h in the dark. The sample was diluted to 1∶10 with 50 mM ammonium bicarbonate and digested with trypsin (20 µg) at 37°C for 18 h. The samples were desalted by solid phase extraction using a STAGE tip protocol (Stop and go extraction tips for matrix-assisted laser desorption/ionization, nano-electrospray, and LC/MS sample pre-treatment in proteomics). The eluted peptides were dried by centrifugal evaporation to remove acetonitrile and redissolved in Solvent A. The resulting peptide mixture was analysed by Liquid Chromatography (LC)/Mass Spectrometry (MS) LC-MS/MS on a 5600 Triple TOF mass spectrometer (AB Sciex, Framingham, U.S.A.) equipped with an Eksigent Nanoflow binary gradient HPLC system and a nanospray III ion source. Solvent A was 0.1% formic acid in water and solvent B was 0.1% formic acid in acetonitrile. MS/MS spectra were collected using Information Dependent Acquisition (IDA) using a survey scan (m/z 350–1500) followed by 25 data-dependent product ion scans of the 25 most intense precursor ions. All mass spectra were analysed using the Mascot and Protein Pilot search engines against the Swissprot-swissprot database with the species set as human (scores greater than 30). False discovery rate (FDR) was estimated using a reversed sequence database. Finally, proteins identified were submitted to bioinformatic pathway analysis (Ingenuity Pathway Analysis [IPA]; Ingenuity Systems, Mountain View, CA; www.ingenuity.com).

### Statistical Analysis

Data are represented as mean ± SEM, with n = 6-12 different cell cultures of CTs isolated from first trimester pregnancies. Comparisons between two group means were performed by unpaired Student's t-test. Multiple groups were compared using the analysis of variance (ANOVA) post hoc analyses were used for pair-wize comparisons (Bonferroni correction test). Statistical significance was defined as p<0.05.

## Results

### Characterization of cytotrophoblast-derived exosomes

**Figure 1:**
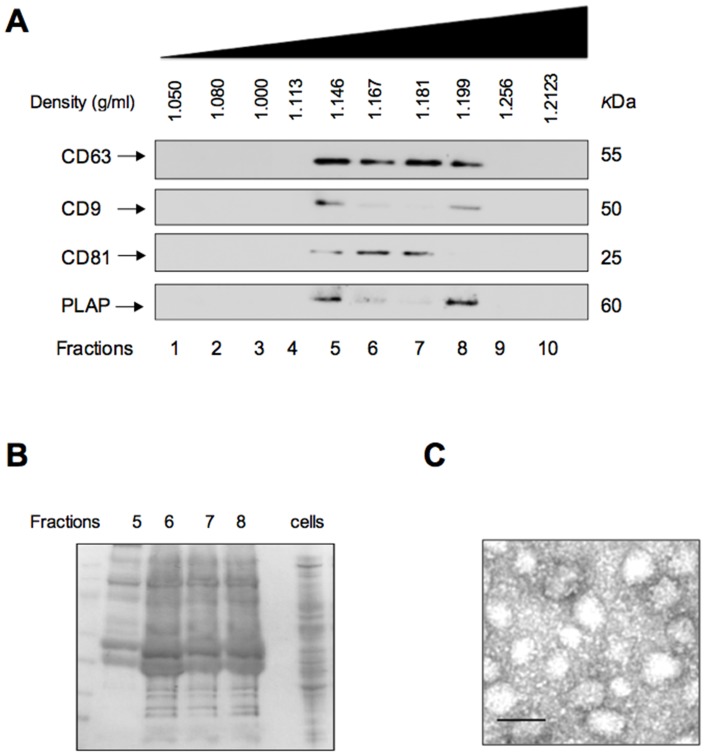
Detection and characterization of Cytotrophoblast cell-derived exosomes. Cytotrophoblast cells were isolated from chorionic villi obtained from first trimester pregnancies and cultured under different oxygen tension (see Methods). Exosomes were isolated from CTs culture media and characterized morphologically and using specific marker for exosome proteins. (A) Representative Western blot for exosome markers: CD63, CD9, CD81 and PLAP. Sample loading was normalized by protein content. Fractions 1 to 10, represent fractions collected after buoyant density centrifugation. (B) Protein profile of exosomal proteins and cytotrophoblast cells proteins. Exosomal proteins derived from fraction 5 to 8 (positive for exosomal marker) and cellular proteins of trophoblast cells (cells) were separated by SDS-PAGE and stained with SimplyBlueTM SafeStain. (C) Electron micrograph of exosomes isolated by ultracentrifuge and purified with a sucrose gradient (pooled exosomal pellet) from cytotrophoblast cells. In B, Scale bar 100 nm. In A, B and C, none of the experiments performed were significantly different using different oxygen tension.

Characterization included analysis of the physical properties of exosomes on a continuous sucrose gradient and protein composition by Western blot. CD63, CD9, CD81 and PLAP positive nano-paticles displayed a buoyant density of 1.146 – 1.199 g/ml (Figure 1A). Each exosome-containing fraction (5 to 8) was separated by SDS-PAGE and stained with SimplyBlue™ SafeStain (Invitrogen). Fractions 5 to 8 displayed a similar protein profile that was distinct from that observed for cytotrophoblast total cellular protein (Figure 1B). The exosomal fractions isolated from CTs were pooled and examined under transmission electron microscopy. Exosomes where identified as vesicles sized between 40–100 nm (Figure 1C).

### Effects of oxygen tension on exosome release

**Figure 2:**
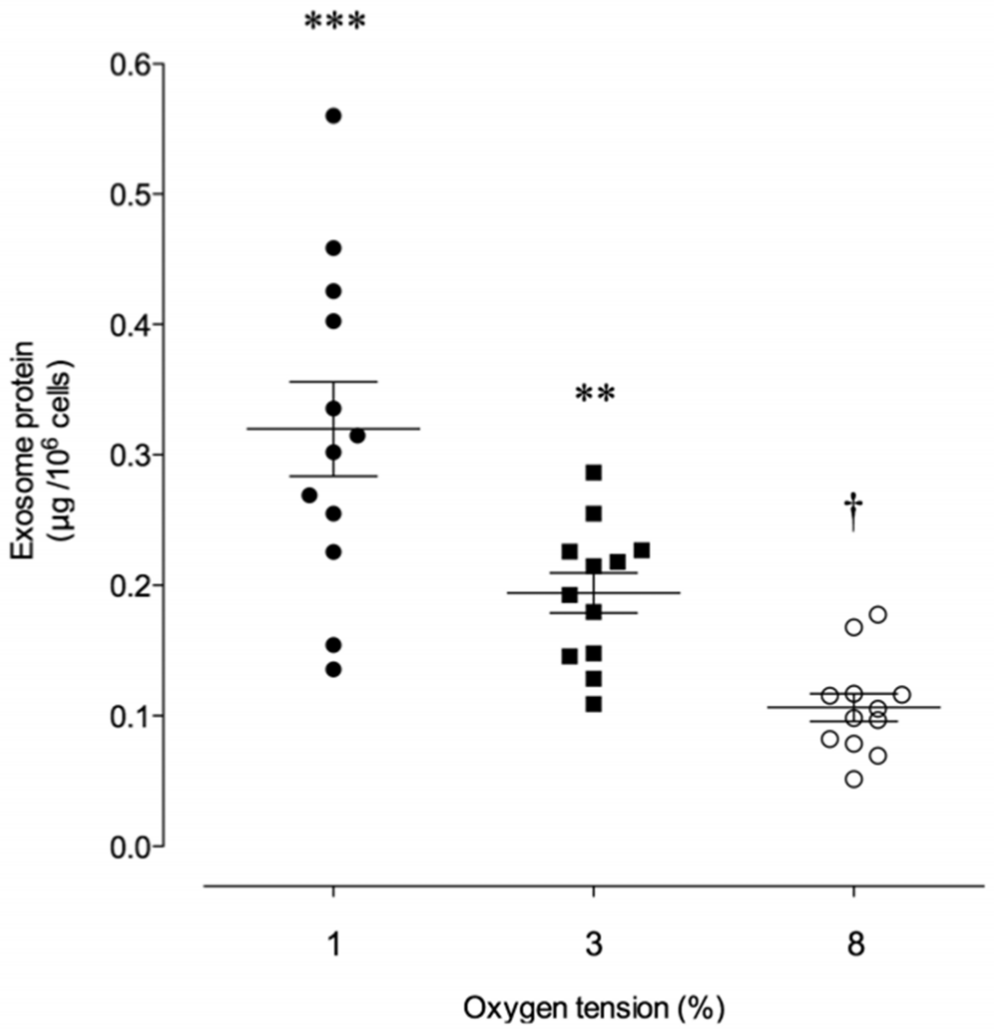
Exosomes released from cytotrophoblast cell exposed to different oxygen tension. Effects of oxygen tension on the release of exosomes from cytotrophoblast cells are presented as ug exosomal protein/106/48 h. Data are presented as a scatter plot with mean ± SEM (n = 6 biological samples and 2 independent duplicate cultures per placenta duplicate). ***p<0.001 versus 8% O2; **p<0.01 versus 1% O2; †p<0.05 versus 3% O2.

The release of exosomes (µg exosomal protein/106 cell/48 h) from CTs was simultaneously assessed at 1%, 3% and 8% O2 using a 3-compartment hypoxia chamber and presented in Figure 2. At 1%, 3% and 8% O2, exosomal protein release averaged 0.32±0.04, 0.19±0.02 and 0.11±0.01 µg protein/106 cells/48 h respectively (ANOVA, p<0.0001, n = 6 in duplicate). Post-hoc tests (Bonferroni's multiple comparison test) identified significant differences between all group means (p<0.05). Cell viability (>95%) and incubation medium pH at 48 h displayed no significant treatment effects (p = 0.80). Incubation chamber oxygen tensions were independently monitored and verified.

### CTs-derived exosomes induce EVT invasion

#### Time course

**Figure 3:**
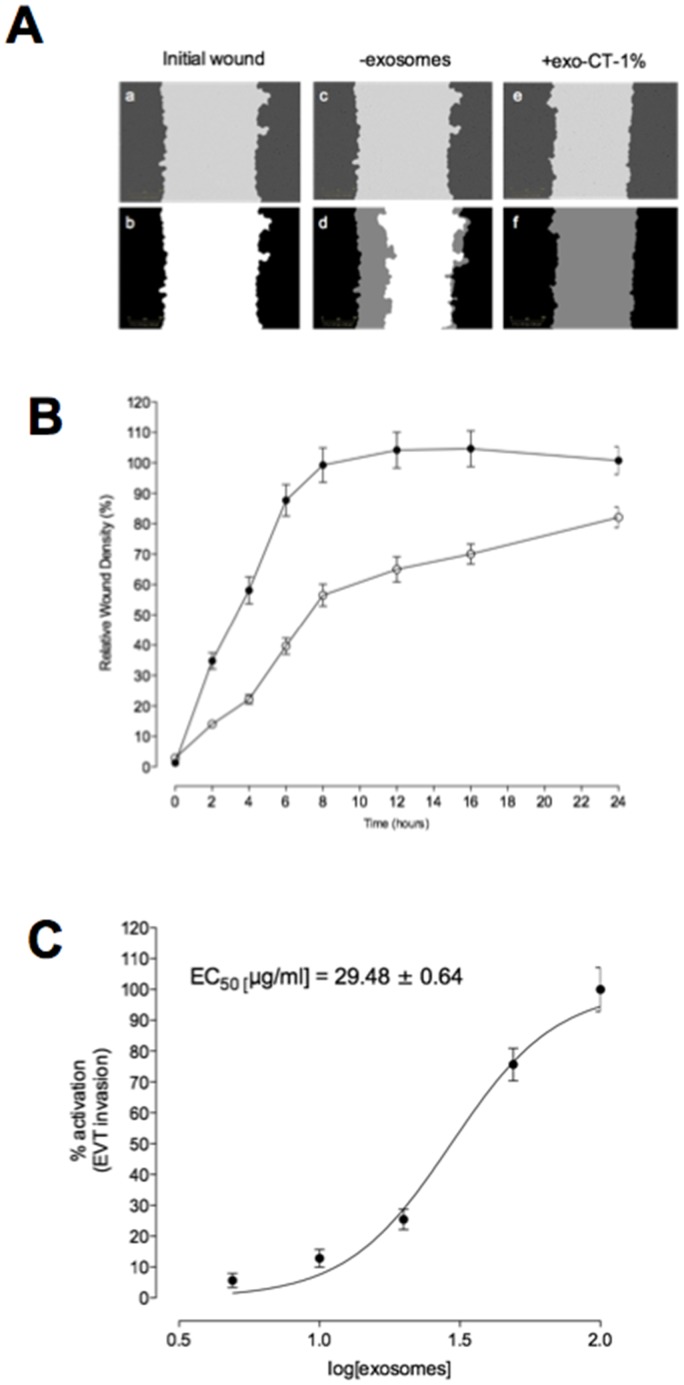
Cytotrophoblast cell-derived exosomes increased EVT invasion. EVT cells were grown to confluence in complete media. A wound was made using 96 well WoundMaker and then overlaid to form a 3D matrix-gel (see Methods). EVT invasion was measured in absence (white circles) or presence (black circles) of 100 ug/ml of exosomes from cytotrophoblast cells exposed to 1% O2 (exo-CTs-1%) for 24 h. (A) Top: a, Wound imaged immediately after wounding; b, Graphical representation from a showing the calculation of initial wound width (black); c and e, Image at the midpoint of the experiment; d and f, Graphical representation from c and e of cell invasion (gray) at the midpoint of the experiment. (B) Time course of wound closure for HTR8/SVneo expressed as relative wound density (%). Data are presented as mean ± SEM for control (no exosomes, open circles) and treatment (100 µg/ml exosomal protein, closed circles). (C) Dose response curve for the effect of CT-derived exosomes on HTR8/SVneo invasion. Data are presented as a non-linear regression analysis (curve fit) and mean ± SEM.

Representative photomicrographs of HTR-8/Svneo wound closure for treatment and control experiments are presented in Figure 3A. The effect of CT-derived exosomes on HTR-8/SVneo cell invasion is presented as relative wound density (percent) over time (Figure 3 B). The rate of wound closure was significantly increased in the presence of CT-derived exosomes as measured by ST50 (9.4±0.4 versus 3.2±0.2, p<0.001) and area under the curves (1333±74 versus 2108±122, p<0.001). The effect of exosomes was concentration dependent (Figure 3C).

#### Dose Response

**Figure 4:**
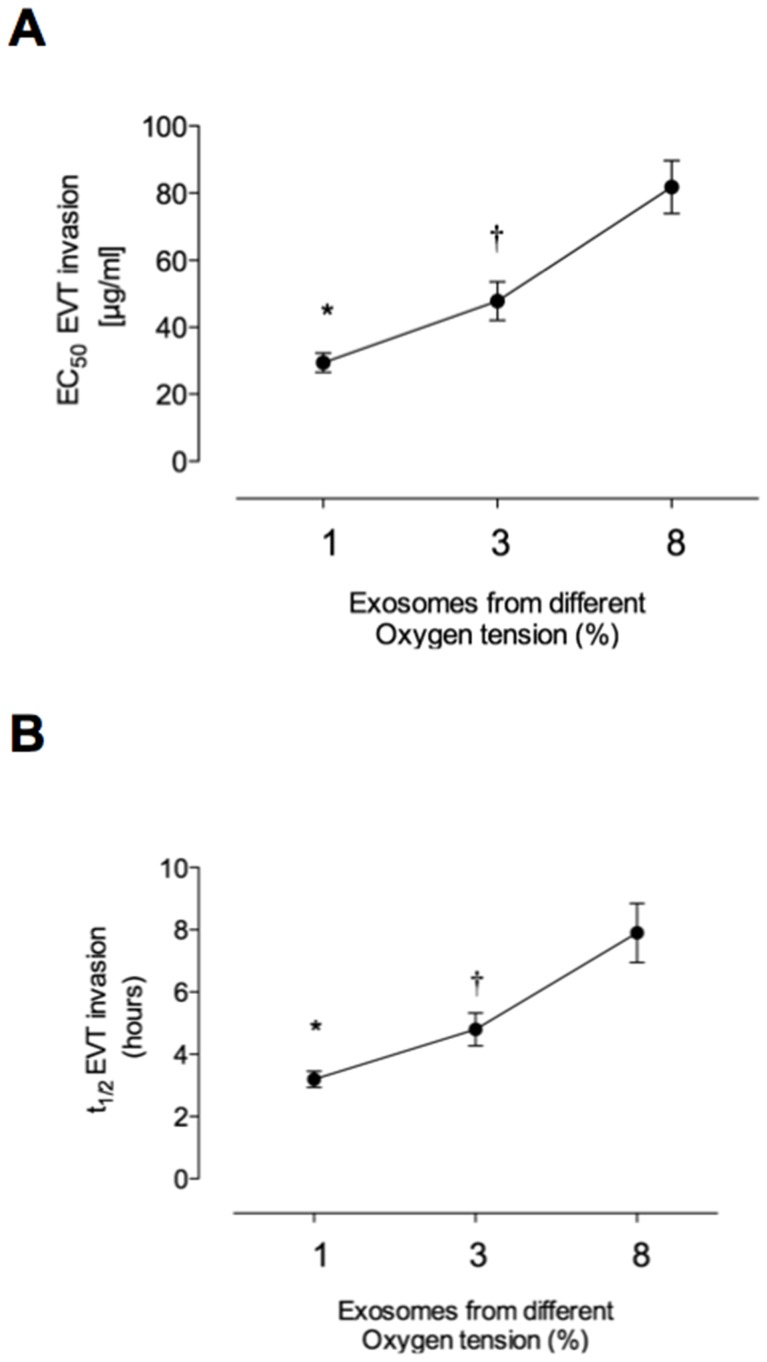
Effect of oxygen tension on exosome bioactivity. EVT cell invasion was measurement in presence of exosomes isolated from cytotrophoblast cells exposed to three different oxygen tension (1%, 3% and 8% O2). (A) The graph represents the changes of half-maximal effective concentration (EC50) and (B) half-maximal stimulatory time (ST50) exosomes on EVT invasion in response to oxygen tension (source). Values are mean ± SEM. *p<0.01 versus all conditions; †p<0.05 versus 8% O2.

The effect of increasing concentrations of CT-derived exosomes on EVT invasion is presented in Figure 4. Exosomes significantly increased HTR-8/SVneo cell invasion and proliferation in a concentration-dependent manner. For cell invasion, EC50 = 29.4±2.1, 47.8±3.2 and 81.8±6.1 µg/ml for treatment in presence of exosomes isolated from 1%, 3% and 8% O2, respectively (p<0.005).

### Effect of oxygen tension on CT-derived exosome bioactivity

In this study, exosome bioactivity was defined as the half maximal effect of exosome on EVT invasion (half-maximal stimulatory time (ST50) and half-maximal effective concentration (EC50)). Exosomes isolated from CTs exposed to 3% (exo-CTs-3%) and 8% O2 (exo-CTs-8%) increased the EC50 on EVT invasion ∼1.6-fold and ∼2.7-fold compared to exo-CTs-1%, respectively (Figure 4A). The effect of different oxygen tension on bioactivity of exosomes was correlated with an increase in the ST50 (∼1.5-fold and ∼2.4-fold with exo-CT-3% and exo-CTs-8%, respectively) compared to effect on EVT invasion in presence of exo-CTs-1% (Figure 4B).

### CTs-derived exosomes increase EVT proliferation

**Figure 5:**
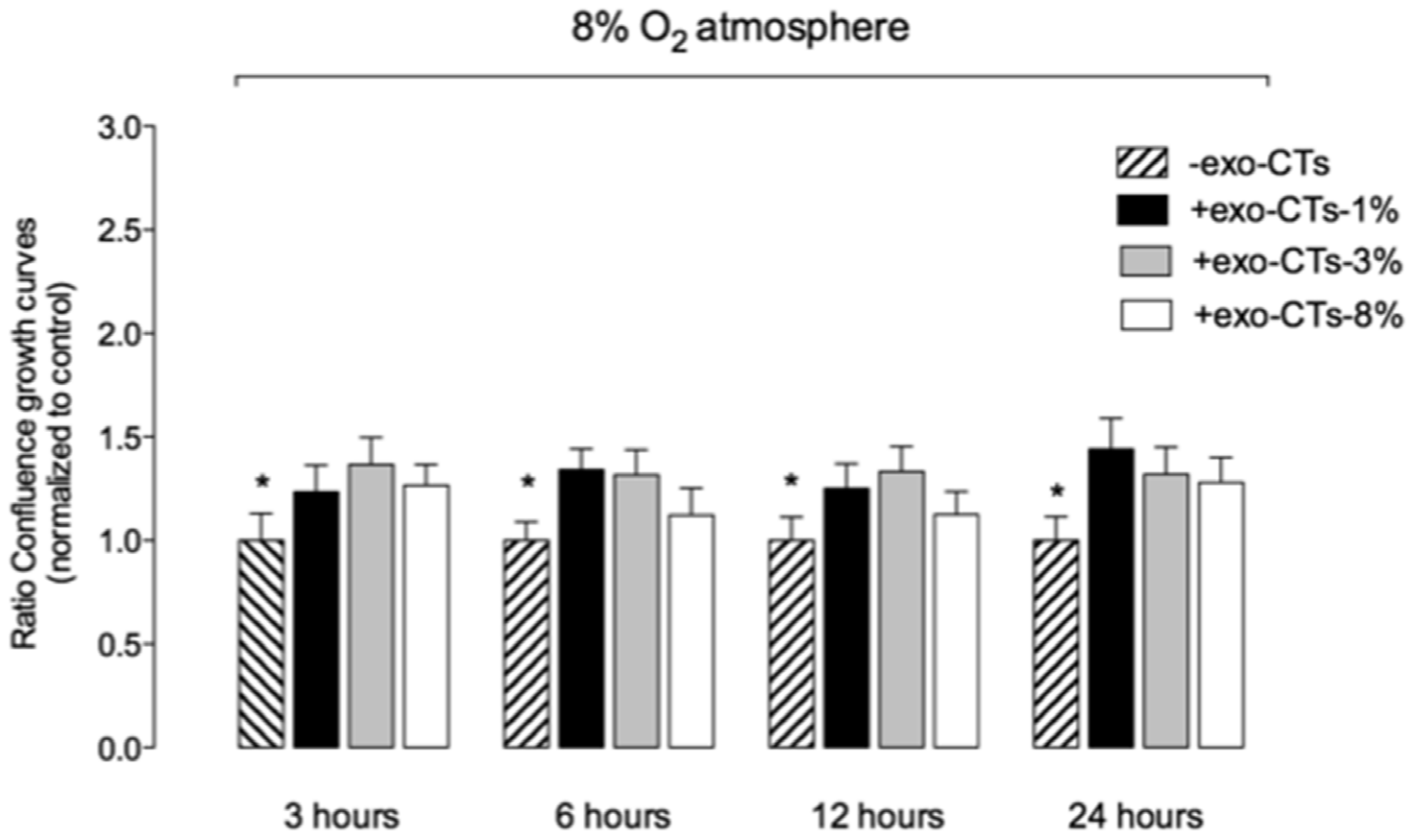
Effect of cytotrophoblast cell-derived exosomes on EVT proliferation. EVT cell proliferation was measurement in presence of exosomes (100 µg/ml) isolated from cytotrophoblast cells exposed to three different oxygen tension (1%, 3% and 8% O2). Values are mean ± SEM. *p<0.01 versus control (-exo-CTs) for each time point.

A real-time imaging system (IncuCyte™) was used to measure cell proliferation using non-label cell monolayer confluence approach. The proliferation ratio (+exosomes/-exosomes for each hour) was significantly higher (p<0.05) compared to the control in the absence of exosomes (Figure 5). At 24 hours, exo-CTs-1% increased EVT proliferation by ∼1.5 fold compared to those in absence of exosomes (control).

### Proteomic analysis of CTs-derived exosomes

**Figure 6:**
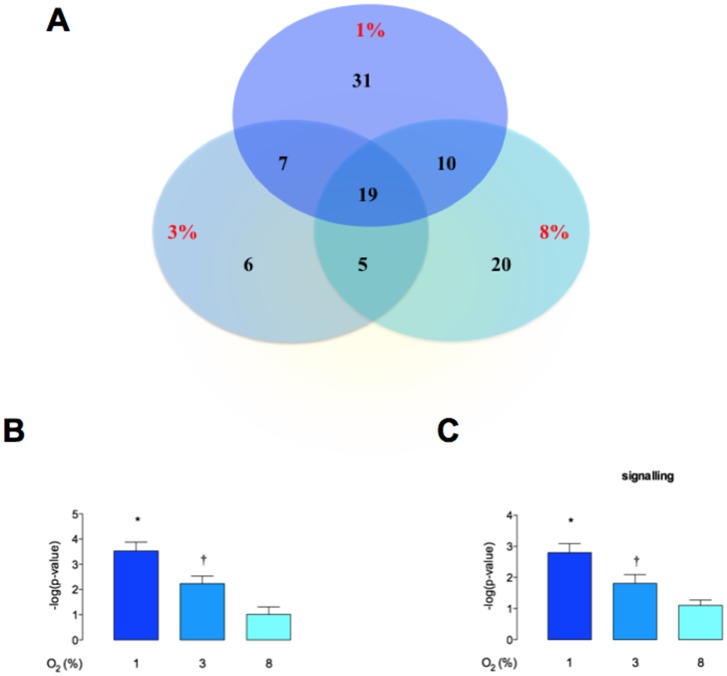
Analysis of cytotrophoblast cell-derived exosome proteins. (A) The Venn diagram represents the distribution of common and unique proteins identified by nanospray LC-MS/MS (ABSciex 5600) in exosomes released from trophoblast cells exposed to 1%, 3% and 8% of oxygen tension. Comparison of canonical pathways: (B) HIFα, and (C) IL-8 signaling identified by IPA core analysis. Values are mean ± SEM. In B and C, *p<0.005 versus all condition; †p<0.05 versus 8% O2.

**Figure 7:**
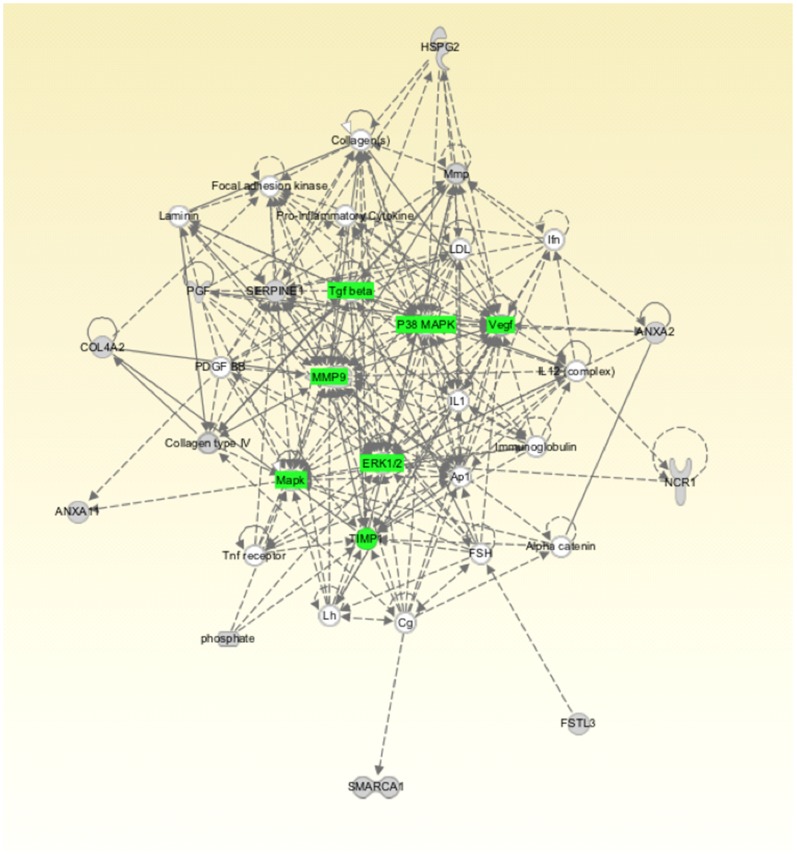
Ingenuity Pathway Analysis of Exosomal Proteins. Unique proteins identified in exosomes isolated from cytotrophoblast cells exposed to 1% oxygen were submitted to IPA network analysis. Green: signaling involving in cellular movement.

Mass spectrometry analysis identified over 160 exosomal proteins (Table 1). We identified unique proteins for each condition (Figure 6). The biological relevance of differentially expression proteins was analyzed using Ingenuity Pathway Analysis (IPA) software. Exosomal proteins isolated from CT exposed to different oxygen tensions were associated with cellular movement and morphology, immune cell trafficking and cellular assembly and organization in accordance with IPA analysis. The canonical pathways associated with our exosomal proteins defined by IPA Core comparison analysis showed that the score (-log [p-value]) for proteins associated with HIF-α signalling (Figure 6B) and IL-8 signalling (Figure 6C) were inversely correlated to oxygen tension. Finally, we investigated the molecular network that can be activated by the unique proteins identified in exosomes isolated from cytotrophoblast cells exposed to 1% O_2_ (31 proteins) (Figure 7). We found molecules involved in cellular movement such as MMP9, TGF-β, P38 MAPK, VEGF and others.

**Table 1:** List of proteins identified in exosomes from CT exposed to different oxygen level.

**1% O2**				
Protein ID	Symbol	Entrez Gene Name	Location	Type(s)
ABHD8_HUMAN	ABHD8	abhydrolase domain containing 8	unknown	enzyme
ACTN1_HUMAN	ACTN1	actinin, alpha 1	Cytoplasm	other
PLCA_HUMAN	AGPAT1	1-acylglycerol-3-phosphate O-acyltransferase 1 (lysophosphatidic acid acyltransferase, alpha)	Cytoplasm	enzyme
ALBU_HUMAN	ALB	albumin	Extracellular Space	transporter
ANXA1_HUMAN	ANXA1	annexin A1	Plasma Membrane	other
ANX11_HUMAN	ANXA11	annexin A11	Nucleus	other
ANXA2_HUMAN	ANXA2	annexin A2	Plasma Membrane	other
APOA1_HUMAN	APOA1	apolipoprotein A-I	Extracellular Space	transporter
CO3_HUMAN	C3	complement component 3	Extracellular Space	peptidase
CO9_HUMAN	C9	complement component 9	Extracellular Space	other
CO4A1_HUMAN	COL4A1	collagen, type IV, alpha 1	Extracellular Space	other
CO4A2_HUMAN	COL4A2	collagen, type IV, alpha 2	Extracellular Space	other
DHX58_HUMAN	DHX58	DEXH (Asp-Glu-X-His) box polypeptide 58	Cytoplasm	enzyme
FBLN1_HUMAN	FBLN1	fibulin 1	Extracellular Space	other
VGFR1_HUMAN	FLT1	fms-related tyrosine kinase 1 (vascular endothelial growth factor/vascular permeability factor receptor)	Plasma Membrane	kinase
FINC_HUMAN	FN1	fibronectin 1	Extracellular Space	enzyme
FSTL3_HUMAN	FSTL3	follistatin-like 3 (secreted glycoprotein)	Extracellular Space	other
G3P_HUMAN	GAPDH	glyceraldehyde-3-phosphate dehydrogenase	Cytoplasm	enzyme
VTDB_HUMAN	GC	group-specific component (vitamin D binding protein)	Extracellular Space	transporter
HBB_HUMAN	HBB	hemoglobin, beta	Cytoplasm	transporter
HBD_HUMAN	HBD	hemoglobin, delta	Cytoplasm	transporter
H2B1B_HUMAN	HIST1H2BB	histone cluster 1, H2bb	Nucleus	other
PGBM_HUMAN	HSPG2	heparan sulfate proteoglycan 2	Plasma Membrane	enzyme
HTRA4_HUMAN	HTRA4	HtrA serine peptidase 4	unknown	other
INS_HUMAN	INS	insulin	Extracellular Space	other
ITIH4_HUMAN	ITIH4	inter-alpha-trypsin inhibitor heavy chain family, member 4	Extracellular Space	other
ITM2B_HUMAN	ITM2B	integral membrane protein 2B	Plasma Membrane	other
K2C1_HUMAN	KRT1	keratin 1	Cytoplasm	other
K1C10_HUMAN	KRT10	keratin 10	Cytoplasm	other
K1C18_HUMAN	KRT18	keratin 18	Cytoplasm	other
K1C19_HUMAN	KRT19	keratin 19	Cytoplasm	other
K2C7_HUMAN	KRT7	keratin 7	Cytoplasm	other
K2C8_HUMAN	KRT8	keratin 8	Cytoplasm	other
LG3BP_HUMAN	LGALS3BP	lectin, galactoside-binding, soluble, 3 binding protein	Plasma Membrane	transmembrane receptor
MMP12_HUMAN	MMP12	matrix metallopeptidase 12 (macrophage elastase)	Extracellular Space	peptidase
MMP2_HUMAN	MMP2	matrix metallopeptidase 2 (gelatinase A, 72kDa gelatinase, 72kDa type IV collagenase)	Extracellular Space	peptidase
MMP9_HUMAN	MMP9	matrix metallopeptidase 9 (gelatinase B, 92kDa gelatinase, 92kDa type IV collagenase)	Extracellular Space	peptidase
MS4A7_HUMAN	MS4A7	membrane-spanning 4-domains, subfamily A, member 7	unknown	other
MYH9_HUMAN	MYH9	myosin, heavy chain 9, non-muscle	Cytoplasm	transporter
NCTR1_HUMAN	NCR1	natural cytotoxicity triggering receptor 1	Plasma Membrane	transmembrane receptor
SLIP_HUMAN	NUGGC	nuclear GTPase, germinal center associated	Nucleus	other
NXF1_HUMAN	NXF1	nuclear RNA export factor 1	Nucleus	transporter
PLGF_HUMAN	PGF	placental growth factor	Extracellular Space	growth factor
PLXB2_HUMAN	PLXNB2	plexin B2	Plasma Membrane	transmembrane receptor
POTEE_HUMAN	POTEE/POTEF	POTE ankyrin domain family, member F	unknown	other
PRG2_HUMAN	PRG2	proteoglycan 2, bone marrow (natural killer cell activator, eosinophil granule major basic protein)	Extracellular Space	other
PSA2_HUMAN	PSMA2	proteasome (prosome, macropain) subunit, alpha type, 2	Cytoplasm	peptidase
PSA6_HUMAN	PSMA6	proteasome (prosome, macropain) subunit, alpha type, 6	Cytoplasm	peptidase
PSA7_HUMAN	PSMA7	proteasome (prosome, macropain) subunit, alpha type, 7	Cytoplasm	peptidase
PSB2_HUMAN	PSMB2	proteasome (prosome, macropain) subunit, beta type, 2	Cytoplasm	peptidase
PSB7_HUMAN	PSMB7	proteasome (prosome, macropain) subunit, beta type, 7	Cytoplasm	peptidase
QSOX1_HUMAN	QSOX1	quiescin Q6 sulfhydryl oxidase 1	Cytoplasm	enzyme
S10AB_HUMAN	S100A11	S100 calcium binding protein A11	Cytoplasm	other
PAI1_HUMAN	SERPINE1	serpin peptidase inhibitor, clade E (nexin, plasminogen activator inhibitor type 1), member 1	Extracellular Space	other
GDN_HUMAN	SERPINE2	serpin peptidase inhibitor, clade E (nexin, plasminogen activator inhibitor type 1), member 2	Extracellular Space	other
SMCA1_HUMAN	SMARCA1	SWI/SNF related, matrix associated, actin dependent regulator of chromatin, subfamily a, member 1	Nucleus	transcription regulator
SYC2L_HUMAN	SYCP2L	synaptonemal complex protein 2-like	Nucleus	other
TRFE_HUMAN	TF	transferrin	Extracellular Space	transporter
TFPI1_HUMAN	TFPI	tissue factor pathway inhibitor (lipoprotein-associated coagulation inhibitor)	Extracellular Space	other
TSP1_HUMAN	THBS1	thrombospondin 1	Extracellular Space	other
TIMP1_HUMAN	TIMP1	TIMP metallopeptidase inhibitor 1	Extracellular Space	other
TINAL_HUMAN	TINAGL1	tubulointerstitial nephritis antigen-like 1	Extracellular Space	transporter
TMEM5_HUMAN	TMEM5	transmembrane protein 5	Plasma Membrane	other
TRRAP_HUMAN	TRRAP	transformation/transcription domain-associated protein	Nucleus	transcription regulator
VAT1_HUMAN	VAT1	vesicle amine transport protein 1 homolog (T. californica)	Plasma Membrane	transporter
WNT7A_HUMAN	WNT7A	wingless-type MMTV integration site family, member 7A	Extracellular Space	cytokine
YIPF1_HUMAN	YIPF1	Yip1 domain family, member 1	Cytoplasm	other
**3% O2**				
Protein ID	Symbol	Entrez Gene Name	Location	Type(s)
ABHD8_HUMAN	ABHD8	abhydrolase domain containing 8	unknown	enzyme
ACTB_HUMAN	ACTB	actin, beta	Cytoplasm	other
ACTN1_HUMAN	ACTN1	actinin, alpha 1	Cytoplasm	other
PLCA_HUMAN	AGPAT1	1-acylglycerol-3-phosphate O-acyltransferase 1 (lysophosphatidic acid acyltransferase, alpha)	Cytoplasm	enzyme
ALBU_HUMAN	ALB	albumin	Extracellular Space	transporter
ASB18_HUMAN	ASB18	ankyrin repeat and SOCS box containing 18	unknown	other
BPTF_HUMAN	BPTF	bromodomain PHD finger transcription factor	Nucleus	transcription regulator
CO4A_HUMAN	C4B (includes others)	complement component 4B (Chido blood group)	Extracellular Space	other
CALI_HUMAN	CCIN	calicin	Cytoplasm	other
CGHB_HUMAN	CGB (includes others)	chorionic gonadotropin, beta polypeptide	Extracellular Space	other
CO4A1_HUMAN	COL4A1	collagen, type IV, alpha 1	Extracellular Space	other
CTND1_HUMAN	CTNND1	catenin (cadherin-associated protein), delta 1	Nucleus	other
VGFR1_HUMAN	FLT1	fms-related tyrosine kinase 1 (vascular endothelial growth factor/vascular permeability factor receptor)	Plasma Membrane	kinase
FINC_HUMAN	FN1	fibronectin 1	Extracellular Space	enzyme
VTDB_HUMAN	GC	group-specific component (vitamin D binding protein)	Extracellular Space	transporter
HBD_HUMAN	HBD	hemoglobin, delta	Cytoplasm	transporter
H2B1B_HUMAN	HIST1H2BB	histone cluster 1, H2bb	Nucleus	other
HTRA4_HUMAN	HTRA4	HtrA serine peptidase 4	unknown	other
INS_HUMAN	INS	insulin	Extracellular Space	other
KIF3C_HUMAN	KIF3C	kinesin family member 3C	Cytoplasm	other
K1C18_HUMAN	KRT18	keratin 18	Cytoplasm	other
K2C8_HUMAN	KRT8	keratin 8	Cytoplasm	other
LG3BP_HUMAN	LGALS3BP	lectin, galactoside-binding, soluble, 3 binding protein	Plasma Membrane	transmembrane receptor
MMP12_HUMAN	MMP12	matrix metallopeptidase 12 (macrophage elastase)	Extracellular Space	peptidase
MMP2_HUMAN	MMP2	matrix metallopeptidase 2 (gelatinase A, 72kDa gelatinase, 72kDa type IV collagenase)	Extracellular Space	peptidase
RM43_HUMAN	MRPL43	mitochondrial ribosomal protein L43	Cytoplasm	translation regulator
NEUL_HUMAN	NLN	neurolysin (metallopeptidase M3 family)	Cytoplasm	peptidase
NXF1_HUMAN	NXF1	nuclear RNA export factor 1	Nucleus	transporter
PLXB2_HUMAN	PLXNB2	plexin B2	Plasma Membrane	transmembrane receptor
GDN_HUMAN	SERPINE2	serpin peptidase inhibitor, clade E (nexin, plasminogen activator inhibitor type 1), member 2	Extracellular Space	other
SNPH_HUMAN	SNPH	syntaphilin	Plasma Membrane	other
SYC2L_HUMAN	SYCP2L	synaptonemal complex protein 2-like	Nucleus	other
TRFE_HUMAN	TF	transferrin	Extracellular Space	transporter
TFPI1_HUMAN	TFPI	tissue factor pathway inhibitor (lipoprotein-associated coagulation inhibitor)	Extracellular Space	other
TSP1_HUMAN	THBS1	thrombospondin 1	Extracellular Space	other
VAT1_HUMAN	VAT1	vesicle amine transport protein 1 homolog (T. californica)	Plasma Membrane	transporter
WNT7A_HUMAN	WNT7A	wingless-type MMTV integration site family, member 7A	Extracellular Space	cytokine
**8% O2**				
Protein ID	Symbol	Entrez Gene Name	Location	Type(s)
A2MG_HUMAN	A2M	alpha-2-macroglobulin	Extracellular Space	transporter
ABHD8_HUMAN	ABHD8	abhydrolase domain containing 8	unknown	enzyme
ADH7_HUMAN	ADH7	alcohol dehydrogenase 7 (class IV), mu or sigma polypeptide	Cytoplasm	enzyme
ALBU_HUMAN	ALB	albumin	Extracellular Space	transporter
ANR17_HUMAN	ANKRD17	ankyrin repeat domain 17	Nucleus	other
ANXA1_HUMAN	ANXA1	annexin A1	Plasma Membrane	other
APOA1_HUMAN	APOA1	apolipoprotein A-I	Extracellular Space	transporter
ASB18_HUMAN	ASB18	ankyrin repeat and SOCS box containing 18	unknown	other
AT2B1_HUMAN	ATP2B1	ATPase, Ca++ transporting, plasma membrane 1	Plasma Membrane	transporter
CO3_HUMAN	C3	complement component 3	Extracellular Space	peptidase
CO4A_HUMAN	C4B (includes others)	complement component 4B (Chido blood group)	Extracellular Space	other
CF118_HUMAN	C6orf118	chromosome 6 open reading frame 118	unknown	other
CGHB_HUMAN	CGB (includes others)	chorionic gonadotropin, beta polypeptide	Extracellular Space	other
CO4A1_HUMAN	COL4A1	collagen, type IV, alpha 1	Extracellular Space	other
CRBA4_HUMAN	CRYBA4	crystallin, beta A4	unknown	other
EF1A1_HUMAN	EEF1A1	eukaryotic translation elongation factor 1 alpha 1	Cytoplasm	translation regulator
EVC_HUMAN	EVC	Ellis van Creveld syndrome	Cytoplasm	other
EZRI_HUMAN	EZR	ezrin	Plasma Membrane	other
FBLN1_HUMAN	FBLN1	fibulin 1	Extracellular Space	other
VGFR1_HUMAN	FLT1	fms-related tyrosine kinase 1 (vascular endothelial growth factor/vascular permeability factor receptor)	Plasma Membrane	kinase
FMN1_HUMAN	FMN1	formin 1	Plasma Membrane	other
FINC_HUMAN	FN1	fibronectin 1	Extracellular Space	enzyme
GKN1_HUMAN	GKN1	gastrokine 1	Extracellular Space	growth factor
HBB_HUMAN	HBB	hemoglobin, beta	Cytoplasm	transporter
HBD_HUMAN	HBD	hemoglobin, delta	Cytoplasm	transporter
H2B1B_HUMAN	HIST1H2BB	histone cluster 1, H2bb	Nucleus	other
HEMO_HUMAN	HPX	hemopexin	Extracellular Space	transporter
ILF3_HUMAN	ILF3	interleukin enhancer binding factor 3, 90kDa	Nucleus	transcription regulator
INS_HUMAN	INS	insulin	Extracellular Space	other
ITIH4_HUMAN	ITIH4	inter-alpha-trypsin inhibitor heavy chain family, member 4	Extracellular Space	other
KI13A_HUMAN	KIF13A	kinesin family member 13A	Cytoplasm	transporter
KIF3C_HUMAN	KIF3C	kinesin family member 3C	Cytoplasm	other
K2C1_HUMAN	KRT1	keratin 1	Cytoplasm	other
K1C18_HUMAN	KRT18	keratin 18	Cytoplasm	other
K2C8_HUMAN	KRT8	keratin 8	Cytoplasm	other
LG3BP_HUMAN	LGALS3BP	lectin, galactoside-binding, soluble, 3 binding protein	Plasma Membrane	transmembrane receptor
MMP2_HUMAN	MMP2	matrix metallopeptidase 2 (gelatinase A, 72kDa gelatinase, 72kDa type IV collagenase)	Extracellular Space	peptidase
MYH9_HUMAN	MYH9	myosin, heavy chain 9, non-muscle	Cytoplasm	transporter
SLIP_HUMAN	NUGGC	nuclear GTPase, germinal center associated	Nucleus	other
PCLO_HUMAN	PCLO	piccolo (presynaptic cytomatrix protein)	Cytoplasm	transporter
PLXB2_HUMAN	PLXNB2	plexin B2	Plasma Membrane	transmembrane receptor
PRG2_HUMAN	PRG2	proteoglycan 2, bone marrow (natural killer cell activator, eosinophil granule major basic protein)	Extracellular Space	other
PSG3_HUMAN	PSG3	pregnancy specific beta-1-glycoprotein 3	Extracellular Space	other
RET4_HUMAN	RBP4	retinol binding protein 4, plasma	Extracellular Space	transporter
SEM4G_HUMAN	SEMA4G	sema domain, immunoglobulin domain (Ig), transmembrane domain (TM) and short cytoplasmic domain, (semaphorin) 4G	Plasma Membrane	other
ANT3_HUMAN	SERPINC1	serpin peptidase inhibitor, clade C (antithrombin), member 1	Extracellular Space	other
GDN_HUMAN	SERPINE2	serpin peptidase inhibitor, clade E (nexin, plasminogen activator inhibitor type 1), member 2	Extracellular Space	other
SNPH_HUMAN	SNPH	syntaphilin	Plasma Membrane	other
SYC2L_HUMAN	SYCP2L	synaptonemal complex protein 2-like	Nucleus	other
TRFE_HUMAN	TF	transferrin	Extracellular Space	transporter
TSP1_HUMAN	THBS1	thrombospondin 1	Extracellular Space	other
YI016_HUMAN	TUBBP5	tubulin, beta pseudogene 5	unknown	other
VAT1_HUMAN	VAT1	vesicle amine transport protein 1 homolog (T. californica)	Plasma Membrane	transporter
WNT7A_HUMAN	WNT7A	wingless-type MMTV integration site family, member 7A	Extracellular Space	cytokine

All mass spectra were analysed using the Mascot and Protein Pilot search engines against the Swissprot-swissprot database with the species set as human (score over 30). Exosomal proteins identified by mass-spectrometry were analyzed with the Ingenuity Pathway Analysis (Ingenuity Systems, www.ingenuity.com). False discovery rate (FDR) was estimated using a reversed sequence database. List of total exosomal protein from cytotrophoblast cells exposed to different oxygen level are presented as Protein ID, Symbol, Entrez Gene Name, Location and type.

## Discussion

Extravillous trophoblast invasion into the maternal tissue is a critical process in placentation. Hypoxia is a risk factor for complications of pregnancy and may adversely affect placentation and development of the materno-fetal vascular exchange. In particular, during early pregnancy low oxygen tension may impact on EVT migration and interactions with the maternal spiral arterioles [24]–[26]. Exosomes from cytotrophoblast cells may interact with EVT and modify their invasiveness, however, the effect and role of exosomes from placental cells has yet to be defined. The aim of this study was to establish the effect of oxygen tension on the release and bioactivity of CT-derived exosomes on EVT invasion and proliferation in vitro. The data obtained are consistent with the hypothesis that exosomes released from cytotrophoblast cells incubated under low oxygen tension promote HTR-8/SVneo invasion and proliferation. While the role of CT exosomes in vivo remains to be established, their release under hypoxic conditions within the placenta may be an adaptive response to promote proliferation and invasion of extravillous trophoblast.

In this study, placental cell exosomes were isolated from cell-conditioned media using differential and buoyant density centrifugation according to our previously published method [10]. CT-derived exosomes displayed: a buoyant density between 1.146 and 1.199 g/ml; positive expression for exosome markers, such as CD63, CD9 and CD81; and a diameter of than less 100 nm analysed under electron microscope. These characteristics are consistent with previously published data [15], [27]. The release of exosomes from CTs was measured as total CD63 positive, particulate protein with a buoyant density 1.146 to 1.199 g/ml.

The release of exosomes from CTs was inversely correlated with oxygen tension. When CTs were incubated under 1% oxygen, exosome release into the incubation medium was 3-fold greater than that observed when cells were incubated under 3% and 8% oxygen. This increase in exosome release was not associated with a loss of cell viability. Our observations that exosome release is affected by oxygen tension are consistent with previously published data obtained from other cell types [28]–[30], including first trimester placental mesenchymal stem cells [10]. While the mechanism(s) by which low oxygen tension increases the release of exosome release remains to be established, oxygen-sensing transcriptional factors, such as HIF-α may be involved [28].

Jauniaux et al., (2000) measured in situ oxygen tension during early pregnancy (at 60 days) within the chorionic placenta, inter-villous space (IVS), and maternal endometrium underlying the placenta [31]. Oxygen tension was ∼3%, 1% and 8%, respectively. Low oxygen tension within the placenta and IVS at this stage of pregnancy may promote the release of exosomes from cytotrophoblast cells and enhance cell-to-cell communication. In particular, exosomes released from CTs may induce functional changes in EVTs that promote cell invasion and proliferation. Consistent with this hypothesis, in this study we demonstrated that CT-derived exosomes increase invasion and proliferation of the EVT cell line HTR-8/SVneo.

During the first trimester of pregnancy, EVT cells invade the decidua and myometrium and regulate the flow of maternal blood into the IVS. EVTs co-localize with maternal spiral arterioles and are present within the lumen of these vessels (where they are thought to prevent flow into the IVS). Subsequent perfusion of the IVS is associated with the transformation of these arterioles from high resistance, low capacity to low resistance, high capacity vessels. EVTs play a role in remodelling these vessels [32], [33]. Abnormal EVT function may result in failure to transform these vessels resulting in compromised placental perfusion and hypoxia [34]. What regulates EVT invasion and/or function and their interactions with maternal vessels remains to be clearly established. The data obtained in this study, however, establish that exosomes released form CTs increase EVT cell invasion in a concentration-dependent manner, and that the activity of exosomes is increased under low oxygen tension.

In this study, HTR-8/SVneo were cultured under low oxygen tension for at least 48 h before experimental manipulation and incubated in the presence of CT-derived exosomes for 24 h. An automated real-time imaging system was used to maintain cells in optimal conditions for quantifying cell invasion and proliferation. Exosome-treatment reduced EVT ST50 compared to control incubations (i.e. absence of exosomes). Furthermore, exosomes isolated from CTs incubated under low oxygen tension (i.e. 1%) displayed greater activity (per unit exosomal protein) than exosomes obtained from cells incubated at higher oxygen tensions (i.e. 3 and 8%). Similar, effects (but of lower magnitude) were observed for EVT cell proliferation.

The effect of low oxygen tension on EVT invasion remains controversial and disparate data have been reported [8], [33], [35], [36]. Studies performed on EVT cells isolated from placental tissue (5 to 10 weeks of gestation) established that low oxygen tension reduces invasion of EVT cells through decreased MMP-2 [36] and appears to be mediated by urokinase plasminogen activator (PLAU) system [8]. In contrast, low oxygen tension (<1% O_2_) increased HTR-8/SVneo cell invasion when compared to cell incubated under 20% O_2_ [37].

The data obtained in the current study establish a role for exosomes in the intercellular communication between placental cells and in regulating EVT cell invasion in an oxygen-dependent manner. Low oxygen tension increased exosome release and modified their protein content. When CTs were incubated under different oxygen tensions, exosomal protein content was altered significantly. Ingenuity Pathway Analysis (IPA) of exosomal proteins identified oxygen-dependent changes in HIFα and IL-8 signalling pathways. In addition, when CTs were incubated under low oxygen tension, exosomal proteins identified were predominantly associated with pathways involved in the activation of MMP-9, TGF-β, MAPK, VEGF, p38MAPK, TIMP1 and ERK1/2. It remains to be established whether or not specific changes in exosomal protein content are causally related to changes in EVT invasion and proliferation. EVT cells have a crucial role in placentation, characterized by their invasion of spiral uterine arteries to establish a low-resistance, high-capacity perfusion system. The mechanism involved in EVT invasion is not completely understood, however, there is a consensus that invading EVT cells up-regulate proteins such as MMPs, integrins (α5β1 and α1β1) and VE-cadherin which support uterine wall invasion. Our data suggest that the exosome released from cytotrophoblast cells also promote EVT invasion including the activation of MMPs, MAPK and invasiveness pathways. We are only beginning to develop an understanding of the role of placental-derived exosomes (*e.g.* cytotrophoblast cells) in early pregnancy events and, in particular, how they might affect the function of key cell-types (*e.g.* EVT) involved in the development of the placenta and its vascular communication with both mother and fetus.

In summary, the release of exosomes from primary culture of cytotrophoblast cells is oxygen tension-dependent. CT-derived exosomes increase EVT cell invasion and proliferation in a concentration and oxygen-dependent manner. Exosomal protein content is altered in response to oxygen tension, with the enhancement of signals involved in cellular invasion and migration. The release of CT-derive exosomes under hypoxic conditions within the placenta may be an adaptive response to promote proliferation and invasion of extravillous trophoblast cells.
